# Anti‐Cancer, Anti‐Inflammatory, and Analgesic Effects of *Taxus wallichaina* Extracts and Its Biosynthesized Silver Nanoparticles

**DOI:** 10.1002/fsn3.71247

**Published:** 2025-11-21

**Authors:** Fazli Hadi, Waqar Ahmad Kaleem, Abdur Rauf, Anees Ahmed Khalil, Huda Ateeq, Ahood Khalid, Naveed Muhammad, Walaa F. Alsanie, Abdulhakeem S. Alamri, Amal F. Alshammary, Muhammad Ibrahim, Mohammed Mansour Quradha

**Affiliations:** ^1^ Department of Pharmacy University of Swabi Swabi Khyber Pakhtunkhwa Pakistan; ^2^ Department of Chemistry University of Swabi Swabi Khyber Pakhtunkhwa Pakistan; ^3^ University Institute of Diet and Nutritional Sciences, Faculty of Allied Health Sciences The University of Lahore Lahore Pakistan; ^4^ University Institute of Food Science and Technology, Faculty of Allied Health Sciences The University of Lahore Lahore Pakistan; ^5^ Department of Pharmacy Abdul Wali Khan University Mardan Khyber Pakhtunkhwa Pakistan; ^6^ Department of Clinical Laboratory Sciences, the Faculty of Applied Medical Sciences Taif University Taif Saudi Arabia; ^7^ Research Center for Health Sciences Taif University Taif Saudi Arabia; ^8^ Department of Clinical Laboratory Sciences, College of Applied Medical Sciences King Saud University Riyadh Saudi Arabia; ^9^ College of Education Seiyun University Seiyun Yemen; ^10^ Pharmacy Department, Medical Sciences Aljanad University for Science and Technology Taiz Yemen

**Keywords:** analgesic, anti‐inflammatory, cytotoxicity, silver nanoparticles, *Taxus wallichiana*

## Abstract

This study investigates the green synthesis of silver nanoparticles (AgNPs) using *Taxus wallichiana Zucc*. and evaluates their pharmacological potential. *Taxus wallichiana*, a medicinal plant rich in bioactive compounds, was utilized to synthesize AgNPs in an eco‐friendly manner, leveraging phytochemicals as reducing and stabilizing agents. Characterization techniques, including UV–Vis spectroscopy confirmed the peak at approximately 430 nm, reaching its maximum at 0.72 with significant surface plasmon resonance peaks. Moreover, SEM identified successful synthesis of stable spherical and oval shapes, with AgNPs ranging in size from 10 to 20 nm, and FTIR exhibited distinct functional groups with peaks at 3649.39, 3622.62, and 3500.05 cm^−1^ as O—H and —CH stretching. Similarly, C≡C stretching was observed at 2300.98 cm^−1^, while the peak at 1570.97 cm^−1^ was associated with the aromatic ring. Comparative analyses of the plant extract and its nanoparticles revealed enhanced anti‐cancer (cytotoxicity up to 85.45%), anti‐inflammatory (reduction up to 0.268 at 120 min), and analgesic (latency ranged from 9.4 to 10.1 s) activities for the AgNPs. Cytotoxicity assays demonstrated that the nanoparticles exhibited superior efficacy against U87 glioblastoma cells compared to the crude extract. Additionally, the study confirmed the safety of both the extract and nanoparticles through acute toxicity tests. While no significant analgesic effects were observed through the Hot plate method, the nanoparticles showed pronounced anti‐inflammatory properties. This research highlights the therapeutic potential of *Taxus wallichiana*‐derived AgNPs and underscores the role of nanotechnology in advancing plant‐based medicine.

## Graphical abstract

*Taxus wallichaina* extracts and its biosynthesized silver nanoparticles shows promising anticancer and anti‐inflammatory properties.

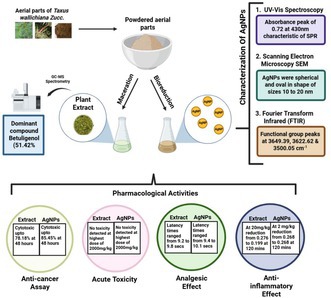

## 1 Introduction

Since centuries, medicinal and herbal plants are known to mankind as an integral part of promoting well‐being and human health (Parveen et al. 2025). Various natural metabolites have served as foundational sources for ancient and modern medicine. According to WHO (World Health Organization), nearly 80% of the world population depends directly or indirectly on ancient medication for their primary health care (Fakih et al. 2022). *Taxus wallichiana* Zucc., commonly known as Himalayan yew in different countries of South Asia like Pakistan, India, and Bangladesh, is classified as a spice due to its unique functional and pharmaceutical properties (Dissanayake et al. 2023).

*Taxus wallichiana* Zucc., belongs to the family Taxaceae that is abundantly present in temperate and subalpine forests of the Himalaya. This medicinal plant is grown in different countries of South‐Eastern Asia such as Nepal, Pakistan, Bhutan, Afghanistan, and India. The leaves and bark of *Taxus wallichiana* Zucc., are considered a cache of diverse secondary constituents, predominantly taxanes. Owing to the presence of these secondary metabolites, the extract of this plant is recognized for its potential antimicrobial, anticancer, antiinflammatory characteristics (Sharma et al. 2020). Even though extracts from different parts of *Taxus wallichiana* Zucc., possess therapeutic potential, its clinical applications still have various challenges like bio‐accessibility and bioavailability of taxanes, limited extraction yield using conventional methods, and the slow growth rate of this plant. Due to these challenges, scientists are focusing on alternative methods including the use of nanotechnology and novel extraction techniques to enhance the bioavailability and extraction yield of bioactive constituents, respectively (Talib et al. 2020).

Species of genus Taxus are characterized in the scientific community as an exceptional plant owing to the presence of unique phytochemicals specifically taxanes. Taxanes are diterpenoid alkaloids, which are utilized as precursors for various drugs used for treating cancer including Taxotere (docetaxel), Taxol (paclitaxel), Jevtana (cabazitaxel). Post FDA approval of paclitaxel back in the 1990s, it is frequently used to treat lung, breast, and ovarian cancers (Sati et al. 2024; Kampan et al. 2015; Fauzee et al. 2011; Jîjie et al. 2025). Owing to its clinical significance and effectiveness as a chemotherapeutic, it is present in the Essential Medicines list of the World Health Organization (WHO) (Swami et al. 2022). Moreover, the extracts from plants belonging to the genus Taxus have strong antioxidant, anti‐inflammatory, antibacterial, and antipyretic properties (Wang et al. 2022; Nisar et al. 2008). Most Taxus species are considered to be a rich source of a diverse array of secondary phytochemicals including steroids, diterpenoid alkaloids, lignins, and flavonoids. Among other species, *Taxus wallichiana* is also considered to be well‐known among the scientific community due to a cache of bioactive compounds specifically diterpenoid alkaloids having strong anticancer properties (Adhikari et al. 2020). Moreover, various other bioactive compounds (steroids, lignans, and flavonoids) have also been reported in different species of genus Taxus including *Taxus wallichiana* (Zhang et al. 2021; Sharma et al. 2022).

Nowadays, scientists are keen on employing nanotechnology, an innovative technique, in fields of cosmetics, agriculture, medicine, and drug‐delivery systems. Various types of nanomaterials such as carbon‐based, metal and metal oxide‐based, lipid‐based, and nanocomposites are being used in medicine and drug‐delivery applications (Narayanan 2024, 2025). Among these, AgNPs, a type of metal‐based nanoparticles, have attained a prominent spot in the scientific community owing to their unique therapeutic and physio‐chemical properties (Ahmed et al. 2016). The synthesis of plant extract‐based AgNPs is considered an environment‐friendly and sustainable approach to traditional physical and chemical methods (Dikshit et al. 2021). The phytochemicals present in plant extracts like terpenes, phenolics, flavonoids, and alkaloids serve as natural reducing agents in the synthesis of AgNPs. The phytochemicals present in plant extracts facilitate in reducing Ag^+^ (silver ions) to Ag^0^ (elemental silver) and increase the therapeutic characteristics of synthesized nanoparticles (Dikshit et al. 2021).

Applications of silver nanoparticles are increasing as antibacterial agents, sensors, in the food industry and as anticancerous agents. Bio logically synthesized silver nanoparticles possess high solubility, stability, high yield and antimicrobial proper ties. The process of AgNPs synthesis is also non‐toxic and cost‐effective (Bawazeer et al. 2021; Sharma et al. 2022).

Plant extract‐based silver nanoparticles synthesized using green methodology reveal increased pharmacological properties as compared to chemically synthesized counterparts, mainly owing to the capped nanoparticles through phytochemicals present in plant extracts (Ivanova et al. 2018).

Furthermore, AgNPs produced by green methods exhibit various significant features, including antibacterial, antioxidant, medical diagnostic, therapeutic, and cytotoxic effects (Sasidharan et al. 2020; Selvam et al. 2025; Majeed et al. 2022).

Established pharmacological importance of unique phytochemicals present in Taxus species led to the selection of *Taxus wallichiana* in this study. The aerial part of this plant encompasses an array of bioactive components like taxoids, terpenes, phenolic acids, flavonoids that serve as an efficient capping agent and bio‐reductant in the development of nanoparticles. Mechanistically, these compounds provide various carbonyl and hydroxyl groups having the capability of reducing Ag^+^ to Ag^0^. Moreover, stabilizing the formulated nanoparticles through hydrogen bonding. In comparison to other green synthesized plant extract‐based nanoparticles, *T. wallichiana* possesses additional advantages as its extract contains anticancer diterpenoids, which may exert synergistic therapeutic potential in nanoparticles developed from this plant extract. Therefore, the green synthesized AgNPs may reveal improved analgesic, anti‐inflammatory, and cytotoxic properties. As compared to physical and chemical methods, the production of green nanoparticles is considered to be environment‐friendly, bio‐suitable, effective, and cost‐efficient; therefore eradicating the use of hazardous reagents while keeping the therapeutic benefits associated with the plant matrix integrated within the nanomaterials. Keeping in view the importance of nanoparticles specifically AgNPs, the current study was designed focusing on the characterization of green synthesized *Taxus wallichiana* plant extract‐based AgNPs owing to its versatile phytochemical profile. The constituent taxanes flavonoids, and alkaloids working as significant reducing and capping agents ensure the formation of stabilized nanoparticles. Moreover, the bio‐synthesized AgNPs were assessed and compared for their synergistically potent therapeutic complex, where the core and the plant jointly enhance the efficacy, thereby portraying their analgesic, anti‐cancer and anti‐inflammatory activities. By integrating traditional botanical knowledge with cutting‐edge nanotechnology, this research aims to develop sustainable nanoparticles for pressing medical and environmental challenges.

## 2 Materials and Methods

### 2.1 Plant Collection

Aerial parts (bark, branches, and leaves) of *Taxus wallichiana* plant were collected with utmost care from Northern Pakistan (Galyat region) in April 2022. A taxonomist, Dr. Muhammad Ilyas, working in the Department of Botany, University of Swabi, Khyber Pakhtunkhwa identifies the collected plant material. To prevent cross‐contamination and moisture accumulation, the collected plant samples were carefully packed in clean and breathable bags before transportation to the laboratory. The process of plant collection was conducted in the month of April, as during this month the weather is optimum for the peak growth of this plant, ensuring maximum content of bioactive constituents.

### 2.2 Plant Extraction

Initially, in the laboratory the plant samples were carefully washed with tap water and later with deionized water to remove dust, dirt, and other foreign contaminants. After washing, the aerial portion was shade‐dried at room temperature to avoid detrimental effects on phytochemical constituents. Upon drying, the dried aerial parts were subjected to a commercial grinder for conversion to fine powder (Naveed et al. 2022). 50 g of powdered aerial part of the plant was later dipped in a hydroethanolic solution (70:30 v/v) and macerated for 12 to 14 days with occasional mechanical shaking for enhanced extraction. Afterwards, the resultant crude extract was subjected to filtration using muslin cloth and Whatman No. 1 filter paper. In the end, the resultant filtrate was subjected to a rotary concentrator at 40°C for collection of semi‐solid extract, which was later stored at 4°C–6°C till further analysis (Batool et al. 2023).

### 2.3 Phytochemical Screening of Plant Extract

#### 2.3.1 Gas Chromatography–Mass Spectrometry (GC–MS) Analysis

GC 2010 spectrometer equipped with PE‐Wax column having internal diameter and film thickness of 60 m × 0.32 mm and 0.25 μm, respectively was employed for GC–MS analysis of *Taxus wallichiana* extract. The carrier gas used in this experimentation was Helium. Initially the chromatographic conditions were 70°C for 5 min followed by an increase in temperature to 120°C at a rate of 2°C/min. Afterwards, the temperature was increased gradually to 240°C at a rate of 3°C/min. The temperature for the injection part was maintained at 250°C, while to allow maximum entry of the sample in the column, a splitless injection mode with 1 min sampling time was used in GC–MS analysis (Sohail et al. 2023; Saleem et al. 2022). Multiple rinsing steps were performed to ensure cleanliness and precision during the injection process. For this purpose, the injection syringe was subjected to three rinses with pre‐ and post‐solvent, together with twice rinsing with the sample. Speeds for suction and injection plunger were set as high and viscosity compensation time was maintained at 0.2 s. The syringe insertion speed was also set to high, and the injection mode was kept in normal operation without employing a terminal air gap. A high‐speed syringe washing system having bulk washing capacity was employed to minimize the chance of cross‐contamination between each sample.

### 2.4 Characterization of Silver Nanoparticles

#### 2.4.1 UV–Visible Spectroscopy

To validate the bio‐synthesis of nanoparticles and investigate their optical characteristics, UV–Vis (Ultraviolet–visible) spectroscopy was performed using a spectrometer (300 Plus Optima, Japan). The analysis showed the occurrence of SPR (surface plasmon resonance) bands that are distinctive characteristics of metallic nanoparticles (Logeswari et al. 2015). Wavelengths of 250–800 nm were used to examine the formation of silver nanoparticles. A characteristic SPR peak was noticed in the range of 350 to 450 nm. For this analysis, quartz cuvettes having a 1 cm long optical path were employed for the spectroscopic analysis of AgNPs.

#### 2.4.2 Scanning Electron Microscopy (SEM)

SEM (scanning electron microscopy) was used for the assessment of morphology, shape, size and surface characteristics of developed nanoparticles. For this, JEM 2100 Jeol CRL Scanning Electron Microscope present at the University of Peshawar, Pakistan was employed to analyze the shape and size of AgNPs by following the protocols of Khattak et al. (2019) and Saeed et al. (2020).

#### 2.4.3 Fourier Transform Infrared (FTIR) Spectroscopy Analysis

Fourier Transform Infrared (FTIR) spectrometer (LABOR FTIR‐990) was employed for identifying the functional groups involved in the bio‐synthesis of AgNPs. The absorption spectra were recorded across the wave number ranging from 4000 to 400 cm^−1^ (Suresh et al. 2016; Azra and Fatima 2024).

### 2.5 Evaluation of Pharmacological Activities

#### 2.5.1 Anti‐Cancer

Briefly, MTT was incubated with a human glioblastoma multiforme (GBM) originated cell line (U87 cell line). To provide optimal conditions for the growth of cells, they were subjected to Dulbecco's Modified Eagle Medium (DMEM) that was augmented with 10% Fetal Bovine Serum (FBS; Thermo Fisher Scientific, Waltham, MA, USA), Penicillin/Streptomycin (1%) and additional glucose. Upon 90% confluence of the cells, they were detached through trypsin and later were sub‐cultured till further analysis.

#### 2.5.2 In Vitro Cytotoxicity Assay (MTT Assay)

The cytotoxic effects of treatments were assessed using the MTT assay on exponentially growing U87 cells. The cells were counted and seeded at a density of 10,000 cells per well in 96‐well plates (Nunc, Roskilde, Denmark), with each well containing 100 μL of medium. All treatment conditions were conducted in triplicate, testing three samples: *T. wallichiana* extract and silver nanoparticles (AgNPs). Stock solutions of the test compounds were prepared in sterile distilled water and diluted into three concentrations: 25.70, 50.75, and 75.66 μg/mL, using cell culture media. The final volume in each well was adjusted to 200 μL. Control wells were included: blank controls containing only media and solvent controls with solvent but no treatment, ensuring that background absorbance could be corrected and any cytotoxic effects attributed solely to the treatments.

After 24 and 48 h of incubation, 15 μL of MTT solution (5 mg/mL in Phosphate Buffered Saline, PBS) was added to each well. The cells were then incubated at 37°C for an additional 3 h. The MTT solution facilitates the production of purple formazan crystals inside metabolically active cells, which can be observed microscopically. These crystals result from the reduction of MTT by NAD(P)H‐dependent oxidoreductase enzymes, reflecting cell viability. Following incubation with MTT, the solution was carefully removed from the wells to avoid disturbing the formazan crystals. To dissolve the crystals, 150 μL of dimethyl sulfoxide (DMSO) (Sigma Aldrich, St. Louis, MO, USA) was added to each well. DMSO acts as a solvent for formazan, producing a purple solution, the intensity of which correlates with the number of viable cells.

The absorbance was measured at 550 nm using a spectrophotometer, quantifying cell viability. Absorbance readings from treated wells were compared with controls to calculate percent viability using the formula:

$$\begin{array}{c} \%\text{Viability}=(A570\;\text{of the treated cells}-A570\;\text{of the control cells}\\ \;-A570\;\text{of the blank cells})\times 100 \end{array}$$

The cytotoxic impact of *T. wallichiana* extract, AgNPs was assessed individually at the three concentrations (25.70, 50.75, and 75.66 μg/mL) after 48 and 72 h of treatment. Throughout the experiment, cell images were captured at different time points and concentrations using a Nikon TS 100 microscope (10× magnification), both before and after MTT addition. These images documented changes in cell morphology and the presence of formazan crystals, providing visual evidence to complement the quantitative results of the MTT assay.

#### 2.5.3 Acute Toxicity

An acute toxicity evaluation was carried out to investigate the effects of *T. wallichiana* crude extract along with synthesized silver nanoparticles. Different dose levels—500, 1000, and 2000 mg/kg body weight—were administered orally (p.o.) to BALB/c mice (*n* = 6 per group). BALB/c mice were distributed into different groups; each group received one separate dose of 500, 1000, and 2000 mg/kg body weight, whereas 10 mL/kg saline was subjected to the control group. Mice were observed closely to see any prominent signs of distress, toxicity, behavioral changes, and physical changes for the first 4 h after administration of each dose. The monitoring procedure was conducted for the next 20 h, while the final assessment was conducted upon completion of 24 h. After the completion of the observation period, the mortality rate within each group was calculated as the percent of animals failing to survive.

### 2.6 Analgesic Effect

#### 2.6.1 Hot Plate Method

The animals were divided into groups, with the negative control group receiving normal saline, the positive control group administered tramadol, and the test groups treated with the *T. wallichiana* extract and its synthesized silver nanoparticles. Thirty minutes after administering the treatments, each animal was placed on a hot plate, and the latency time (in seconds) was recorded. The latency measurements were taken at different time intervals (30, 60, 90, and 120 min). The following formula was used for the calculation of percentage analgesic effect:

$$\%\text{Analgesic effect}=\frac{\text{latency time of test}-\text{latency time of control}}{\text{cutoff time}-\text{latency time of control}}\times 100$$

#### 2.6.2 Anti‐Inflammatory Activity (Carrageenan‐Induced Paw Edema)

An anti‐inflammatory activity assay was conducted using mice of both sexes, each weighing between 25 and 30 g. The animals were randomly divided into eight groups (*n* = 6). Group I served as the negative control and received normal saline (10 mL/kg), while Group II was the positive control and was treated with diclofenac sodium (10 mg/kg). Groups III to VIII were administered the *T. wallichiana* extract and its synthesized silver nanoparticles at doses of 10 and 20 mg/kg for plant extract and at doses of 1 and 2 mg/kg for silver nanoparticles respectively.

To induce inflammation, carrageenan (1%, 0.05 mL, subcutaneously) was injected into the subplantar tissue of the right hind paw of each mouse 30 min after treatment administration. The anti‐inflammatory response was assessed at intervals of 0, 30, 60, 90, and 120 min using a plethysmometer (LE 7500, Plan Lab S.L.). The degree of anti‐inflammatory activity was expressed as the percentage inhibition of edema, calculated using the following formula.

$$\%\text{Inhibition}=\frac{A-B}{B}\times 100$$

In the formula, “*A*” denotes the edema volume of the negative control group, while “*B*” represents the paw edema observed in the treated group.

## 3 Results

### 3.1 Gas Chromatography–Mass Spectrometry (GC–MS) Analysis

The GC–MS analysis of the plant extract identified a wide range of bioactive compounds with distinct retention times and concentrations. Betuligenol emerged as the most dominant compound, accounting for 51.42% of the total extract. Other notable constituents included 3‐(p‐Hydroxyphenyl)‐1‐propanol (10.96%), Methy‐(2‐hydroxy‐3‐ethoxy‐benzyl)ether (6.33%), and n‐Hexadecanoic acid (5.68%). Additionally, significant amounts of 2‐Butanone, 4‐(4‐hydroxyphenyl)‐ (4.76%) and 2,3‐Bis(1‐methylallyl)pyrrolidine (4.24%) were also identified. Minor components such as Decane, Undecane, Benzenecarbolic acid, 9‐Borabicyclo[3.3.1]nonan‐9‐ol, and Oleic acid, methyl ester were detected, alongside trace levels of fatty acids like Linoleic acid, methyl ester, and Tetradecanoic acid as displayed in Table 1. This detailed analysis highlights the presence of a mixture of aliphatic hydrocarbons, phenolic derivatives, fatty acids, and other organic compounds within the extract, potentially contributing to its biological properties.

**TABLE 1:** Quantitative results of GC–MS analysis of *T. wallichiana* extract.

S. no	Name	Retention time (min)	Area under curve (volt‐minute)	Conc. (%)
1	Decane	7.212	142,316	0.95
2	Undecane	9.588	80,936	0.54
3	Benzenecarbolic acid	11.801	569,465	3.82
4	9‐Borabicyclo[3.3.1]nonan‐9‐ol	18.968	368,622	2.47
5	3‐(p‐Hydroxyphenyl)‐1‐propanol	19.877	1,633,566	10.96
6	2‐Butanone, 4‐(4‐hydroxyphenyl)—	20.003	710,092	4.76
7	Betuligenol	20.438	7,665,371	51.42
8	Methy‐ (2‐hydroxy‐3‐ethoxy‐benzyl) ether	21.791	943,794	6.33
9	Coniferyl alcohol, dehydrodi—	22.214	128,383	0.86
10	Tetradecanoic acid	23.761	33,934	0.23
11	2,3‐Bis(1‐methylallyl)pyrrolidine	24.465	631,982	4.24
12	2‐Aminoformanilide	26.343	14,803	0.1
13	Dimethyl 2‐O‐methylhexopyranosiduronate	26.517	573,348	3.85
14	n‐Hexadecanoic acid	27.236	846,978	5.68
15	Tetratriacontane	27.629	99,405	0.67
16	Cyclotetracosane	29.068	62,305	0.42
17	Linoleic acid, methyl ester	29.234	128,675	0.86
18	Oleic acid, methyl ester	29.332	289,353	1.94

### 3.2 UV–Visible Spectroscopy

**FIGURE 1:**
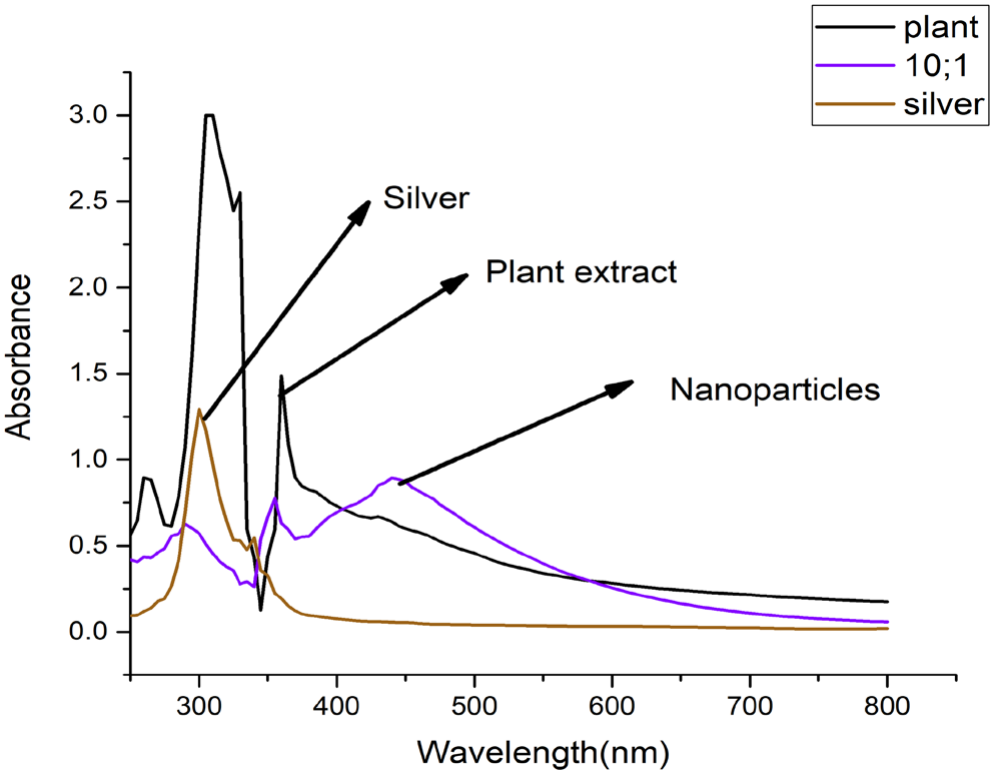
UV–Vis absorbance graph for *T. wallichiana* extract and synthesized AgNPs.

The UV–Vis spectroscopy results revealed that the *λ*_max_ of the plant‐synthesized AgNPs fell within the 400–550 nm range. For *T. wallichiana*‐derived nanoparticles, the *λ*_max_ values for AgNPs were observed at 430 and 433 nm, respectively, with maximum absorbance values of 0.720 and 0.813 (Figure 1). A significant absorbance peak was noted at approximately 430 nm, reaching its maximum at 0.72. This peak is characteristic of the surface plasmon resonance (SPR) associated with silver nanoparticles, a phenomenon where conduction electrons on the nanoparticle surface resonate with the electromagnetic field of incident light, resulting in strong absorption at a specific wavelength. The absorbance values outside the SPR peak ranged from 0.05 to 0.12, both before and after the peak, specifically between 370–420 and 440–500 nm. This indicates minimal light absorption in these regions, suggesting that the nanoparticles exhibit negligible absorbance outside the SPR peak (Figure 1).

### 3.3 Scanning Electron Microscopy (SEM)

**FIGURE 2:**
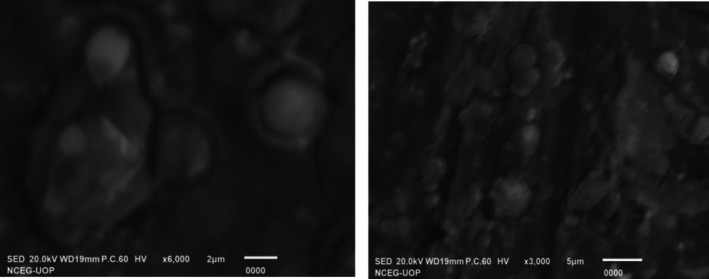
SEM micrograph (6000× and 3000×) for *T. wallichiana* extract‐based AgNPs.

To examine the morphology, surface characteristics, and stability of AgNPs, scanning electron microscopy (SEM) was performed at magnifications of 700×, 1000×, 1300×, 2200×, 3000×, 5500×, and 6000× for AgNPs. The SEM images revealed that the plant extracts act as both reducing agents and capping agents, facilitating the formation of relatively stable, monodispersed nanoparticles with a spherical shape. The particle morphology, stability, and distribution were found to be greatly affected by the experimental parameters and the precursor used for the bioreduction process. The synthesized nanoparticles primarily exhibited spherical and oval shapes, with AgNPs ranging in size from 10 to 20 nm as shown in Figure 2.

### 3.4 Fourier Transform Infrared (FTIR) Spectroscopy Analysis

**FIGURE 3:**
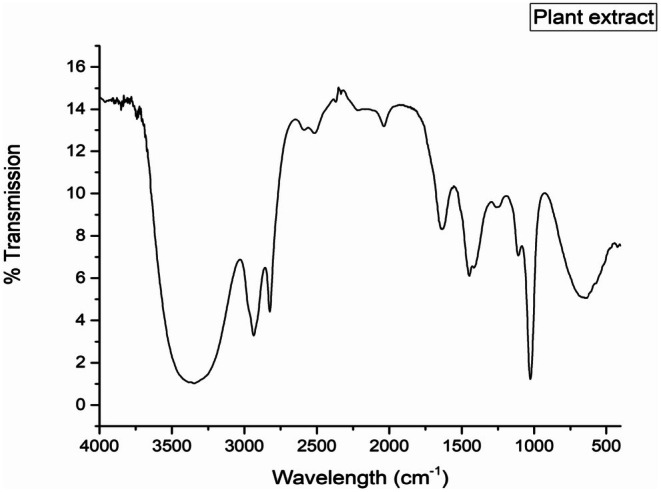
FT‐IR spectrum for *T. wallichiana* plant extract.

The FTIR analysis of the plant extract revealed distinct absorption bands indicative of various functional groups. Broad O‐H stretching vibrations, associated with free hydroxyl groups, were observed in the range of 3267–3500 cm^−1^. C—H stretching vibrations appeared within 2940–2851 cm^−1^, while an additional O—H stretching band was detected between 2584 and 2521 cm^−1^. A prominent peak corresponding to N=C=S stretching was identified at 2039 cm^−1^. Furthermore, C=C stretching vibrations were observed at 1634 cm^−1^, and N—O asymmetric stretching was noted at 1544 cm^−1^. The analysis also identified C—C stretching at 1437 cm^−1^, C—O stretching at 1027 cm^−1^, and C—Br stretching at 642 cm^−1^, reflecting the diverse functional groups present in the sample, as illustrated in Figure 3.

**FIGURE 4:**
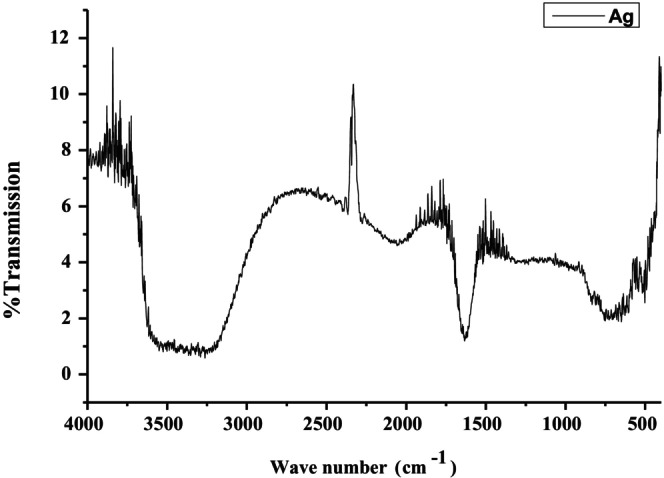
FT‐IR spectrum for *T. wallichiana‐*based AgNPs.

The AgNPs synthesized using *T. wallichiana* plant extract exhibited distinct functional groups. Peaks at 3649.39, 3622.62, and 3500.05 cm^−1^ were indicative of O—H and —CH stretching. C≡C stretching was observed at 2300.98 cm^−1^. The peak at 1570.97 cm^−1^ was associated with the aromatic ring, while the band at 630.09 cm^−1^ suggested the presence of either a para‐disubstituted aromatic group or C—H stretching in an alkyne, as shown in Figure 4.

### 3.5 Anticancer Assay

The cytotoxic activity of *T. wallichiana* extract and silver nanoparticles (AgNPs) against U87 glioblastoma cells was assessed over a 24‐h and 48‐h period using an MTT assay, as described in the methodology. The results are tabulated in Table 2.

**TABLE 2:** The cytotoxicity of *T. wallichiana* extracts and silver nanoparticles on U87 glioblastoma cells.

Sample	Dose (μg/mL)	24 h	48 h
Plant extract	25.70	14.65	65.87
	50.75	16.02	72.65
	75.66	22.32	78.18
Silver NPs	25.32	26.98	66.12
	50.09	32.11	78.32
	75.09	44.32	85.45

The cytotoxicity of *T. wallichiana* plant extract and its silver nanoparticles on U87 glioblastoma cells was evaluated at different doses over 24 and 48 h. At a dose of 25.70 μg/mL, the plant extract showed a cell viability of 14.65% at 24 h, which increased to 65.87% at 48 h. At a dose of 50.75 μg/mL, cell viability was 16.02% at 24 h and 72.65% at 48 h, while at 75.66 μg/mL, it was 22.32% at 24 h and 78.18% at 48 h. For the silver nanoparticles, at a dose of 25.32 μg/mL, cell viability was 26.98% at 24 h and 66.12% at 48 h. At 50.09 μg/mL, cell viability was 32.11% at 24 h and 78.32% at 48 h, and at 75.09 μg/mL, the cell viability decreased to 44.32% at 24 h and 85.45% at 48 h. Overall, the silver nanoparticles exhibited greater cytotoxic effects than the plant extract at all doses, with the effect becoming more pronounced after 48 h of exposure.

### 3.6 Acute Toxicity

The results of the acute toxicity study indicated that both the crude plant extract and the synthesized silver nanoparticles were safe at all tested doses of 500, 1000, and 2000 mg/kg when administered orally. During the 24‐h observation period following treatment, no adverse effects or abnormal behaviors were observed in the test animals, as outlined in Table 3. The absence of toxicity or any visible side effects, even at the highest dose of 2000 mg/kg, strongly supports the safety of these substances.

**TABLE 3:** Acute toxicity of *Taxus walliciana* crude extract and its prepared silver nanoparticles.

Samples	Dose (mg/kg)	Gross effect after 4 h	Mortality after 24 h
Plant extract	500	—	—
	1000	—	—
	2000	—	—
Silver NPs	500	—	—
	1000	—	—
	2000	—	—

### 3.7 Analgesic Effect (Hot Plate Method)

The results from the hot plate method showed no significant variation in latency times across all treatment groups when compared to the control shown in Table 4. The group receiving normal saline had an average latency of 9.5 ± 1.2 s, which was consistent with baseline measurements. Neither the hydroalcoholic plant extract nor its synthesized silver nanoparticles (AgNPs) led to a notable increase in latency times at either of the doses (10 and 20 mg/kg). The latency times for both doses of the extract ranged from 9.2 ± 1.4 s to 9.8 ± 1.6 s, similar to the values observed in the saline‐treated group. Similarly, the silver nanoparticles did not exhibit any analgesic effects, with latency times ranging from 9.4 ± 1.3 s to 10.1 ± 1.5 s. No significant changes were observed at any time point, indicating that there was no central analgesic action observed in any of the groups.

**TABLE 4:** Analgesic effects of *Taxus walliciana* extract and its green sysnthesized AgNPs.

Analgesic effect (Hot Plate Method)						
Nalaxone dose = 0.08 mg						
Compound	Dose (mg/kg)	0 min	30 min	60 min	90 min	120 min
NS	10 ml	9.20 ± 0.01	9.22 ± 0.21	9.16 ± 0.01	9.20 ± 0.02	9.12 ± 0.03
Tramadol	20	9.21 ± 0.20	25.34 ± 0.32**	25.88 ± 0.21***	25.80 ± 0.34***	25.77 ± 0.28***
Plant extract	10	20.01 ± 0.30	17.94 ± 0.25	23.36 ± 0.28	14.65 ± 0.80	10.78 ± 0.60
	20	11.00 ± 0.80	14.35 ± 0.23	25.29 ± 0.42	26.01 ± 0.80	20.02 ± 0.36
Silver NPs	1	21.09 ± 0.28	17.62 ± 0.62	10.45 ± 0.82	20.99 ± 0.47	20.21 ± 0.62
	2	13.00 ± 0.85	16.05 ± 0.25	11.48 ± 0.47	14.50 ± 0.29	14.90 ± 0.84

*Note:* Analgesic effects are presented as Mean ± SEM. Two‐way analysis of variance was conducted at *p* < 0.05 followed by Tuckey's HSD for mean‐pairwise comparison.

** *p* < 0.01.

*** *p* < 0.001.

### 3.8 Anti‐Inflammatory Effect

The anti‐inflammatory effects of the whole plant of *Taxus wallichiana* and its synthesized silver nanoparticles were assessed at various time intervals, as shown in Table 5. The normal saline (NS) control group showed a steady value of 0.321 ± 0.65 at the initial time point, with minor fluctuations observed across all time intervals. In contrast, diclofenac (10 mg) demonstrated significant reductions in inflammation, with values of 0.311 ± 0.65 at 0 min and decreasing progressively to 0.170 ± 0.52 at 120 min, showing statistically significant differences at all time points.

**TABLE 5:** Anti‐inflammatory effect of whole plant of *Taxus walliciana*, and its synthesized silver nanoparticles.

Time	Dose (mg/kg)	0 min	30 min	60 min	90 min	120 min
NS	10 mL	0.321 ± 0.65	0.311 ± 0.25	0.315 ± 0.64	0.236 ± 0.35	0.320 ± 0.52
Diclofenac	10	0.311 ± 0.65	0.201 ± 0.25***	0.225 ± 0.64***	0.136 ± 0.35***	0.170 ± 0.52***
Plant extract	10	0.311 ± 0.65	0.301 ± 0.25	0.225 ± 0.64***	0.136 ± 0.35***	0.180 ± 0.52***
	20	0.333 ± 0.40	0.264 ± 0.56*	0.225 ± 0.50***	0.220 ± 0.80***	0.199 ± 0.70***
Silver NPs	1	0.315 ± 0.36	0.288 ± 0.51**	0.163 ± 0.22***	0.122 ± 0.50***	0.210 ± 0.37***
	2	0.304 ± 0.81	0.256 ± 0.30**	0.180 ± 0.50***	0.150 ± 0.45***	0.268 ± 0.60***

*Note:* Anti‐inflammatory effects are presented as Mean ± SEM. Two‐way analysis of variance was conducted at *p* < 0.05 followed by Tuckey's HSD for mean‐pairwise comparison.

* *p* < 0.05.

** *p* < 0.01.

*** *p* < 0.001.

For the plant extract, at a dose of 10 mg/kg, the inflammation level at 0 min was 0.311 ± 0.65, which gradually decreased to 0.180 ± 0.52 at 120 min. The 20 mg/kg dose of the plant extract exhibited a similar trend, starting at 0.333 ± 0.40 and declining to 0.199 ± 0.70 at 120 min, with significant reductions observed at later time points. The silver nanoparticles (AgNPs) at a 1 mg dose showed a decrease in inflammation, with the value at 0 min being 0.315 ± 0.36, and it reduced to 0.210 ± 0.37 at 120 min. Similarly, the 2 mg dose of AgNPs showed a reduction from 0.304 ± 0.81 at 0 min to 0.268 ± 0.50 at 120 min, with significant effects noted at multiple intervals. Overall, the results indicate that both the plant extract and its silver nanoparticles demonstrated a dose‐ and time‐dependent anti‐inflammatory effect, with diclofenac showing the most pronounced reduction in inflammation across all time points.

## 4 Discussion

The genus Taxus or Yew holds immense importance globally from a pharmacological point of view. A lot of research work has been undertaken to explore the pharmacological importance of the genus. The findings highlight the diverse pharmacological potential of *Taxus wallichiana* and its silver nanoparticles (AgNPs) through various analyses. The GC–MS analysis identified a wide array of bioactive compounds in the plant extract, with Betuligenol being the most abundant (51.42%), alongside other notable compounds like 3‐(p‐Hydroxyphenyl)‐1‐propanol, Methy‐(2‐hydroxy‐3‐ethoxy‐benzyl)ether, and n‐Hexadecanoic acid. These compounds, known for their biological activities, emphasize the therapeutic potential of *T. wallichiana* and its traditional medicinal uses. UV–Visible spectroscopy confirmed the successful synthesis of AgNPs, with characteristic surface plasmon resonance peaks at 430–433 nm, indicating nanoparticle formation and stability. The position and intensity of these SPR bands provided insights into the size and concentration of the nanoparticles (Rauf et al. 2021; Rajasekharreddy et al. 2010). The well‐defined and consistent SPR peaks in the UV–Vis spectrum confirmed the successful formation of nanoparticles within the colloidal suspension, indicating their stability.

Results of our current study are in line with earlier findings of Bhusari et al. (2023). They reported that the addition of plant extract (*Taxus wallichiana* Zucc.) into silver nitrate solution (1 mM) resulted in color change from colorless to dark brown solution. UV–Vis's spectrum showed the formation of AgNPs in the resultant mixture as a clear peak was noticed between 420 and 450 nm. In our current study, a significant absorbance peak was noticed ranging from 420 to 440 nm. A monodisperse size distribution is suggested based on the sharp symmetry of the peaks. Furthermore, the surface plasmon resonance peak between 420 and 440 nm, confirms the colloidal stability of developed nanoparticles (Valsalam et al. 2019).

SEM analysis further revealed the AgNPs' spherical and oval morphologies, with sizes ranging from 10 to 20 nm, demonstrating the extract's effectiveness as a reducing and capping agent. On the other hand, Yousaf et al. (2022) revealed the formation of spherical‐shaped silver oxide particles having sizes varying from 200 to 1000 nm. Moreover, the outcomes of the present study revealed that FTIR spectroscopy identified functional groups, such as phenolic and hydroxyl groups, involved in the reduction and stabilization of nanoparticles, highlighting the eco‐friendly and efficient synthesis process. FTIR spectra in our study matched bands for different functional groups as reported by Adhikari et al. (2023). FTIR spectra for synthesized AgNPs in this study, revealed peaks at 3649.39, 3622.62, 3500.05, 2300.98, 1570.97, and 630.09 cm^−1^ for distinct functional groups and these are in accordance with already published literature (Rashmi et al. 2020; Mandal et al. 2021; Afreen et al. 2020; Gopinath et al. 2015; Yousaf et al. 2022). FTIR analysis of plant extract and biosynthesized nanoparticles helps in identifying the molecules responsible for reduction and stabilization.

After the synthesis of nanoparticles, a shift of O—H stretching from 3267 to 3500 cm^−1^ and the C=C stretching vibrations observed at 1634 cm^−1^ are linked with the reduction of Ag^+^ ions and capping of nanoparticles due to flavonoids and terpenoid carbonyl groups (Baharara et al. 2014).

Synthesized nanoparticles show significant anticancer properties due to increased surface area to volume proportion and their potential to gradually release the chemotherapeutic agents. Nanoparticles deliver potent cytotoxic capabilities when synthesized for tumor targeting, hence, reducing the likelihood of systemic toxicity and enhancing targeted delivery of chemotherapeutic agents in the tumor (Xu et al. 2020). Broadly, the mechanism of action involves the generation of reactive oxygen species and disruption of cancer cell membrane and DNA, therefore efficiently targeting various types of cancers. Moreover, the stability of synthesized nanoparticles is affected by different factors like surface chemistry, shape, size, particle aggregation, electrostatic interactions, stabilization, and interaction with biological systems (Cavallo et al. 2015). Similarly, solvent properties and surface modifications also impart a significant role in the stability of synthesized nanoparticles (Santhiya Selvam et al. 2025; Narayanan 2024).

Recently, interest in the synthesis of silver nanoparticles using different plant extracts has increased owing to their sustainability, eco‐friendly, and cost‐effective nature (Van Phu et al. 2014). Literature shows the significance of plant extract‐based silver nanoparticles as potential antimicrobial, antiinflammatory, and anti‐cancer agents (Salve et al. 2022). Various methods like photochemical treatment, laser mediation, and electro‐irradiation are usually employed for the synthesis of silver nanoparticles; however, due to their expensive and toxic nature, plant extract‐based biological synthesis of nanoparticles is preferred (Zia et al. 2016; Srirangam and Rao 2017). Biosynthesis of AgNPs prepared using different plant extracts possesses various advantages like reduced toxicity, sustainability, and even particle size distribution (Islam et al. 2019; Naganthran et al. 2022; Van Phu et al. 2014). Moreover, plant extracts comprise various secondary constituents, which possess reducing properties; hence they play an important role in the stabilization of silver nanoparticles (Bhusari et al. 2023).

To date, cancer is considered one of the prominent public health concerns owing to its mortality rate and toxic effects of medical therapies like radio‐ and chemo‐therapies. Currently, few plant‐derived compounds like taxol, vinblastine, and vincristine have been employed in modern medicine for their significant anticancer properties (Koul et al. 2021). Synthesized plant extract‐based AgNPs increase the oxidative stress in cancer cells through generation of excessive Reactive Oxygen Species (ROS), which result in disruption of MMP (mitochondrial membrane potential) and help in releasing pro‐apoptotic factors like cytochrome‐c, hence commencing the intrinsic apoptotic pathway (Bhusari et al. 2023). The anticancer properties of plant‐based AgNPs against several cancer cell lines such as U251, MDA‐MB‐231, and MCF‐7 have been reported in the literature (Saranya and Geetha 2014). Moreover, their potential against the SMMC‐7721 cancer cell line has also been reported (Tahir et al. 2020). Similarly, the present cytotoxicity study demonstrated that both the plant extract and AgNPs exhibited dose‐ and time‐dependent activity against U87 glioblastoma cells, with the nanoparticles showing superior efficacy. This enhanced cytotoxicity is likely due to their nanoscale properties, such as increased surface area and cellular penetration. Acute toxicity evaluations revealed no adverse effects at doses up to 2000 mg/kg, establishing a strong safety profile for both the extract and nanoparticles. Analgesic activity testing using the hot plate method indicated that neither the extract nor the nanoparticles exhibited significant central pain‐relieving effects, suggesting limited interaction with opioid receptors or central pain pathways (Sidhu and Mangal 2024).

**FIGURE 5:**
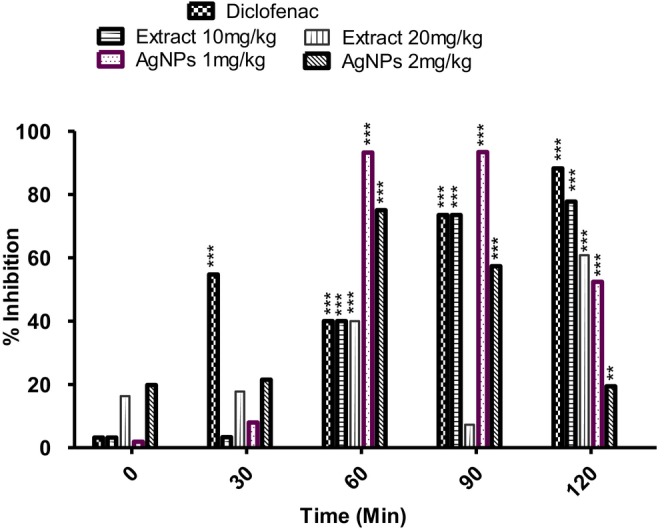
Percent anti‐inflammatory effect of Diclofenac (10 mg/kg), *Taxus walliciana* crude extract at the doses of 10 and 20 mg/kg, its synthesized AgNPs at doses of 1 and 2 mg/kg. *p* < 0.05*, *p* < 0.01**, *p* < 0.001***.

Likewise, inflammation is considered to be linked with the onset of various medical complications like atherosclerosis, diabetes, asthma, cancer, rheumatoid arthritis, etc. (Sinha 2020). AgNPs and plant extracts have shown promising anti‐inflammatory properties (Bhusari et al. 2023). According to a study, during inflammation, *T. wallichiana* AgNPs (100–500 μg mL^−1^) have been found to be promising in protecting lysis in red blood cell membranes. This anti‐inflammatory effect of AgNPs was due to the stabilizing effect that actually prevented leakage of serum proteins from RBCs (Rajeshkumar et al. 2021). The significant anti‐inflammatory properties of synthesized nanoparticles may be due to the inhibition of prostaglandin synthesis and COX‐2, showing the potential of plant phenols delivered through synthesized AgNPs. Moreover, the plant extract and synthesized AgNPs down‐regulate the activation of Nuclear Factor kappa B (NF‐kappa B), hence reducing the transcription of inflammatory genes (Nisar et al. 2008). Synthesized AgNPs had the potential to act as an anti‐inflammatory agent through the protection of RBC membranes from hemolysis (Prathyusha et al. 2018). Likewise, in this study, both the extract and its AgNPs demonstrated notable anti‐inflammatory effects, with dose‐ and time‐dependent reductions in inflammation, although diclofenac showed the highest efficacy. The improved anti‐inflammatory activity of AgNPs compared to the crude extract highlights the potential of nanoparticle formulations in enhancing bioavailability and therapeutic outcomes. Overall, the results for anti‐inflammatory potential for both plant extract and its silver nanoparticles are comparable to that of the standard drug (diclofenac) showing a dose‐ and time‐dependent anti‐inflammatory effect in reducing inflammation across all time points (Table 5 and Figure 5).

As compared to plant extract, the synthesized *Taxus wallichiana* extract‐based AgNPs showed momentous effectiveness in all the biological activities examined in this study. The results show an increase in viability at 48 h for plant extract (78.18%), whereas in the case of AgNPs the viability was noticed as 85.45%. Similarly, a reduction in inflammation due to plant extract‐based silver nanoparticles was documented to be 37.31% as compared to plant extract (27.90% reduction). The significant reduction in inflammation and increase in viability show that synthesized AgNPs augmented the biological activities of plant extract, mainly due to increased surface area of synthesized AgNPs and bio‐capped molecules from the plant extract (Yousaf et al. 2022). These findings collectively suggest that *T. wallichiana* and its silver nanoparticles possess significant pharmacological properties, particularly in oncology and inflammation‐related conditions, while maintaining a favorable safety profile. Future studies should further investigate their mechanisms of action and therapeutic potential in clinical applications.

## 5 Conclusions

Current study revealed the bio‐synthesis of *Taxus wallichiana* Zucc., extract‐based silver nanoparticles, demonstrating a sustainable and environment‐friendly method for developing nanomaterials. Developed AgNPs showed distinctive optical and structural properties as visible from their spherical and oval shapes with AgNPs ranging in size from 10 to 20 nm. Notably, pharmacological analyses showed that synthesized AgNPs had significant cytotoxic potential against U87 glioblastoma cells, along with distinct anti‐inflammatory characteristics. Even though the analgesic potential was less prominent, synthesized nanoparticles performed better as compared to crude plant extracts. Moreover, plant extract and synthesized AgNPs were found to be safe as confirmed by acute toxicity studies, signifying their therapeutic viability. In summary, the outcomes of the present study focus on the integration of nanotechnology with plant extracts for the production of bioactive nanomaterials having promising pharmaceutical properties. Further studies must be conducted to analyze the mechanism of action and evaluate the long‐term safety profiles before moving forward with clinical and pharmaceutical trials of bio‐synthesized AgNPs. Nevertheless, further studies must be designed focusing on molecular docking and simulation analysis along with in vivo pharmacokinetics to validate and fully examine the potential of synthesized nanoparticles.

## Conflicts of Interest

The authors declare no conflicts of interest.

## Data Availability

The data that support the findings of this study are available from the corresponding authors upon reasonable request.
